# High Efficiency Regeneration System from Blueberry Leaves and Stems

**DOI:** 10.3390/life13010242

**Published:** 2023-01-15

**Authors:** Yangyan Zhou, Qing Li, Zejia Wang, Yue Zhang

**Affiliations:** 1 School of Advanced Agricultural Sciences, Peking University, Beijing 100871, China; 2 Key Research Institute of Yellow River Civilization and Sustainable Development & Collaborative Innovation Center on Yellow River Civilization, Henan University, Kaifeng 475001, China; 3 Grassland Agri-Husbandry Research Center, College of Grassland Science, Qingdao Agricultural University, Qingdao 266109, China

**Keywords:** blueberry, leaf regeneration, stem regeneration, efficient breeding, industrial seedling

## Abstract

The main propagation approach is tissue culture in blueberries, and tissue culture is an effective and low-cost method with higher economic efficiency in blueberries. However, there is a lack of stable and efficient production systems of industrialization of tissue culture in blueberries. In this study, the high-efficiency tissue culture and rapid propagation technology system were established based on blueberry leaves and stems. The optimal medium for callus induction was WPM (woody plant medium) containing 2.0 mg/L Forchlorfenuron (CPPU), 0.2 mg/L 2-isopentenyladenine (2-ip) with a 97% callus induction rate and a callus differentiation rate of 71% by using blueberry leaves as explants. The optimal secondary culture of the leaf callus medium was WPM containing 3.0 mg/L CPPU with an increment coefficient of 24%. The optimal bud growth medium was WPM containing 1.0 mg/L CPPU, 0.4 mg/L 2-ip, with which the growth of the bud was better, stronger and faster. The optimal rooting medium was 1/2 Murashige and Skoog (1/2MS) medium containing 2.0 mg/L naphthylacetic acid (NAA), with which the rooting rate was 90% with shorter rooting time and more adventitious root. In addition, we established a regeneration system based on blueberry stems. The optimal preculture medium in blueberry stem explants was MS medium containing 2-(N-morpholino) ethanesulfonic acid (MES) containing 0.2 mg/L indole-3-acetic acid (IAA), 0.1 mg/L CPPU, 100 mg/L NaCl, with which the germination rate of the bud was 93%. The optimal medium for fast plant growth was MS medium containing MES containing 0.4 mg/L zeatin (ZT), 1 mg/L putrescine, 1 mg/L spermidine, 1 mg/L spermidine, which had a good growth state and growth rate. The optimal cultivation for plantlet growth was MS medium containing MES containing 0.5 mg/L isopentene adenine, with which the plantlet was strong. The optimal rooting medium for the stem was 1/2MS medium containing 2.0 mg/L NAA, with which the rooting rate was 93% with a short time and more adventitious root. In conclusion, we found that stem explants had higher regeneration efficiency for a stable and efficient production system of industrialization of tissue culture. This study provides theoretical guidance and technical support in precision breeding and standardization and industrialization in the blueberry industry.

## 1. Introduction

Blueberry (Vaccinieae, Vaccinioideae, Ericaceae) is a perennially deciduous or evergreen shrub. Blueberry fruit is rich in anthocyanins, unsaturated fatty acids, calcium, potassium, zinc, iron and other elements [1]. Blueberry is listed as one of the top five health foods by the International Food and Agriculture Organization [1], and blueberry is known as the king of fruit, which will become one of the most promising fruit tree species in the world in the future [1]. In recent years, with increased understanding of the health function of blueberry fruit, the demand for blueberry in fruit consumption has steadily increased [2], which has greatly promoted the rapid expansion of the blueberry industry, especially in the blueberry seedling and planting industry [3,4]. Therefore, there is an urgent need for rapid production of blueberry seedlings.

With the continuous improvement of the market demand for blueberries, the planting scale and area continue to expand, and the yield has increased substantially. The propagation rate and coefficient of blueberries by cutting are slow and low, and cutting is vulnerable to the degradation of variety in blueberries. The cutting has been difficult to supply in a timely manner to the market with high-quality and consistent seedlings in the blueberry market. Plant tissue culture originated from the concept of totipotency of plant cells, which was proposed by Haberlandt in 1905 [5].

Plant tissue culture is a technology in which the tissue, organ and cell isolated from plant are cultured aseptically in a medium containing nutrient and plant hormones, and the whole plant is regenerated [6].

Plant tissue culture has many significant advantages in the breeding of seedlings, including high propagation rate, high quality and efficiency and no time restrictions. Therefore, plant tissue culture has been widely used in the production of blueberry seedlings [7]. However, many factors have influenced the establishment of the regeneration system in blueberries [8,9,10,11,12,13,14].

The main factors are listed as follows: 1. Variety of blueberries. The plant tissue culture system of different varieties in plants makes a great difference [15]. 2. Explant is a key factor in the establishment of a regeneration system in blueberries, which directly affects the efficiency of the establishment of a regeneration system [16]. Explants should not only take into account the types of organs and tissues from which the explants are obtained, but also the size and physiological age of the explant [17]. In general, young leaves, young buds, stem tip meristem and root tip are used as explants in the establishment of a regeneration system [18]. The explant should be easy to disinfect and should have a high regeneration rate on a suitable medium [19]. 3. The composition of the medium is a key factor that affects success and efficiency in plant tissue culture. The composition of the medium mainly includes major elements, trace elements, iron salts, sucrose, vitamins, plant growth hormone and so on. In the study of plant tissue culture, the selection of a basic medium is given priority, including MS, 1/2MS, LS, B5, N6, WPM medium [20,21,22,23,24,25]. Similarly, plant hormones play an important role in plant tissue culture [26]. In addition, sucrose has a close relationship with the osmotic pressure of the medium, which affects the differentiation and growth in plant tissue [27]. Therefore, the medium composition should be determined by plant species, explant type and growth stage. Taken together, our results show a high efficiency regeneration system from leaves and stems in blueberries.

## 2. Materials and Methods

### 2.1. Selection and Treatment of Explant

The explants were obtained from the young leaves (without petiole) and shoots (without leaf) of a healthy 2-year-old JE blueberry plant, which were planted in the nursery of Shandong Salver group (Rizhao, China). The explants were rinsed under running tap water for 6 min and then rinsed five times with sterilized ddH_2_O, drained with sterile absorbent paper, sterilized on a super-clean work table with 75% (*v*/*v*) alcohol for 20 s, followed by immersion with 2% (*v*/*v*) NaClO for 5 min to 8 min, respectively. Finally, the sterilized explants were watered with sterile water and inoculated on the medium. The growth of explants was observed, and the survival rate was calculated.

### 2.2. Medium for Tissue Culture in Blueberry

#### 2.2.1. Medium for Induction of Callus Based on Leaf

The WPM medium [24] was used as the base medium, which contained 25 g/L sucrose, 6.5 g/L agar and related hormones. Finally, the pH of the medium was adjusted to 5.2. The combination and concentration of hormones are shown in Table 1. In the callus culture of the 18 treatments, the morphological changes in the callus induction of leaves in blueberry explants were statistically analyzed, including callus growth, time of callus appearance, callus morphology, callus color, and callus induction rate and differentiation rate.

#### 2.2.2. Medium for Subculture of Callus Based on Leaf

The WPM medium was used as the base medium, which contained 25 g/L sucrose, 6.5 g/L agar and related hormones. Finally, the pH of the medium was adjusted to 5.2. The concentration of hormones is shown in Table 2.

#### 2.2.3. Medium for Growth of Bud Based on Leaf

The WPM medium was used as the base medium, which contained 25 g/L sucrose, 6.5 g/L agar and related hormones. Finally, the pH of the medium was adjusted to 5.2. The combination and concentration of hormones are shown in Table 3.

#### 2.2.4. Medium for Generation of Root in Bud Based on Leaf

The 1/2MS medium was used as the base medium, which contained 25 g/L sucrose, 6.5 g/L agar and related hormones. Finally, the pH of the medium was adjusted to 5.2. The concentration of hormones is shown in Table 4.

#### 2.2.5. Medium for Pre-Culture of Stem

The MS medium containing MES was used as the base medium [20], which contained sucrose, agar, NaCl and related hormones. Finally, the pH of the medium was adjusted to 5.2. The hormones and substances are shown in Table 5.

#### 2.2.6. Medium for a Rapid Culture of Stem

The MS medium containing MES was used as the base medium, which contained 30 g/L sucrose and 7 g/L agar. Finally, the pH of the medium was adjusted to 5.2. The hormones and substances are shown in Table 6.

#### 2.2.7. Medium for Culturing Seedling of Stem

The MS medium containing MES was used as the base medium, which contained 30 g/L sucrose, 7 g/L agar. Finally, the pH of the medium was adjusted to 5.2. The concentration of isopentene adenine is shown in Table 7.

#### 2.2.8. Medium for Rooting Culture of Stem

The 1/2MS medium was used as the base medium, which contained 20 g/L sucrose, 6.0 g/L agar and NAA. Finally, the pH of the medium was adjusted to 5.2–5.4. The concentration of NAA is shown in Table 8

### 2.3. Establishment of Regeneration System Based on Leaves in Blueberry

#### 2.3.1. Induction of Callus from Leaves

(a) Make 2–3 diagonal cuts in the direction perpendicular to the main vein, while making sure to cut the blade at the same time. Puncture the leaves 3–5 times with pointed tweezers to better facilitate callus induction. One sterile filter paper was replaced for every 10 leaves;
(b) The front of the treated leaves in (a) was placed on the medium upward, and the back of the leaves was fully in contact with the medium, leaving a gap of about 0.5 cm between the leaves, which provides a sufficient growth space for the leaves;
(c) The above petri dishes were sealed and placed in a dark culture chamber at 24 °C for 14–28 days, during which the callus differentiation rate was calculated;
(d) The culture dishes were removed from the dark culture chamber and cultured at 24 °C in 16 h/8 h light/darkness for 14–28 days at the light intensity of 1500–3000 lux to induce bud differentiation and calculate the bud induction rate.

#### 2.3.2. Callus of Leaves Subculture

The callus with better and more robust growth were placed on a sterile ultra-clean workbench, and then cut into squares with the size of 0.5 × 0.5 cm with a sterile scalpel. Then, they were placed in the culture medium for the callus subculture for subculture proliferation. The culture dish was placed in the dark culture room at 24 °C for 3–4 weeks to observe the proliferation state of the callus and calculate the induction rate of the callus.

#### 2.3.3. Bud from Leaves Growth

After dark culture in Section 2.3.2, the callus with better growth, strong growth and undifferentiated buds were transferred to the bud induction medium, which was cultured at 24 °C in 16 h/8 h light/darkness for 15–20 days, and the light intensity was 1500–3000 lux. The growth state of the buds was observed. The induction rate of the adventitious buds from callus regeneration was calculated.

#### 2.3.4. Root Growth Based on Leaves

After cutting the seedlings in Section 2.3.3, which were respectively transferred to the four kinds of rooting medium with 8–10 plants per bottle, they were cultured at 24 °C in 16 h/8 h light/darkness for 20–30 days at a light intensity of 1500–3000 lux. To calculate the rooting rate of tissue culture seedlings in the blueberries, we randomly selected 15–20 bottles of rooting plants for statistics of the rooting rate. Root rate = (number of rooted plants/total plant number) × 100%.

### 2.4. Establishment of Regeneration System Based on Stem in Blueberry

#### 2.4.1. Pre-Culture of Explants from Stem

Stems were placed on a stem pre-culture medium. The interval between the stem was not less than 0.5 mm, and the culture was maintained in darkness at 16 °C for 2 h (2–4 h). Subsequently, the culture medium was placed in 16 h/8 h light/darkness with light intensity of 1500–3000 lux and relative humidity of 60% for 2 d culture.

#### 2.4.2. Rapid Culture of Stem

After the pre-culture, the stem was transferred to the rapid culture medium. The interval between the stem was not less than 0.5 mm. They were cultured at 24 °C in 16 h/8 h light/darkness with light intensity of 1500–3000 lux and relative humidity of 60% for 15–20 days.

#### 2.4.3. Growth Culture of Stem

After the rapid culture, the stem was transferred to the seedling growth medium. The culture medium was placed at 24 °C in 16 h/8 h light/darkness with light intensity of 1500–3000 lux and relative humidity of 60%.

#### 2.4.4. Induction of Root Based on Stem

After cutting the well-growing and robust seedlings in Section 2.4.3 into small segments, they were moved to the rooting medium. The plants were cultured at 24 °C in 16 h/8 h light/darkness with light intensity of 1500–3000 lux for 20–30 days. To calculate the rooting rate of the tissue culture seedlings in the blueberries, we randomly selected 15–20 bottles of rooting plants for statistics of the rooting rate. Root rate = (number of rooted plants/total plant number) × 100%.

### 2.5. Plant Refining and Transplanting

Remove the rooted blueberry seedlings from the bottle, remove the medium, and wash the roots with water before transferring them to a nutrient bowl. The culture soil used for transplanting is vermiculite: nutrient soil: moss (1:1:1), and the pH of nutrient soil should be controlled at 5.3–5.6. The plants were placed in a greenhouse with a humidity of 70% for seedling cultivation. When the root system of blueberry seedlings grew over the paper bowl, they were moved from the greenhouse to the field for cultivation through the integration of water and fertilizer [28,29].

## 3. Results

### 3.1. Induction System of Leaf Regeneration

#### 3.1.1. Induction of Callus from Leaf in Blueberry

In the induction of the callus experiment, there were 18 groups (Table 9). The statistical result of callus growth, time of callus appearance, callus morphology, callus color, and callus induction rate and differentiation rate showed that the callus induction rate and differentiation rate had the best effect, and the difference was obvious under WPM + 2.0 mg/L CPPU + 0.2 mg/L 2-ip conditions (Table 9).

**Figure 1:**
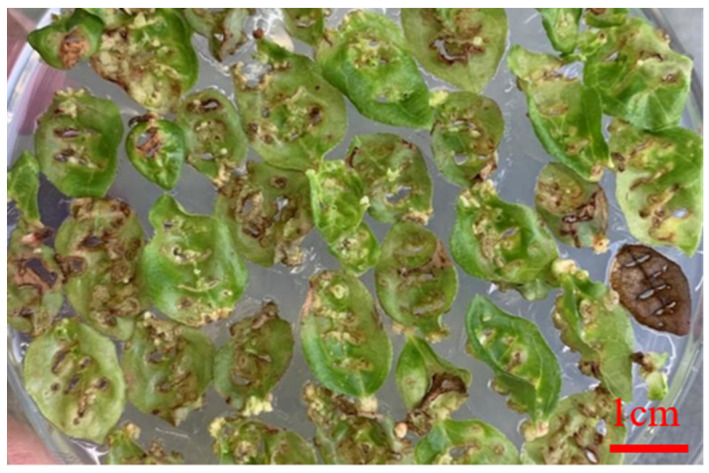
Phenotype of leaf-induced callus by differentiation in blueberry. Bar, 1 cm.

Further observation showed that WPM + 2.0 mg/L CPPU + 0.2 mg/L 2-ip has the best effect on callus induction, which could effectively and rapidly induce callus formation in blueberry leaves. The callus was compact and spherical with a small surface and few particles, most of which were light white or light yellow. The induction rate of the callus was 97%, and the differentiation rate of the callus was 71%. The callus changed from yellow to light yellow, the texture of the callus was soft and the growth rate was accelerated (Figure 1).

#### 3.1.2. Subculture of Callus from Leaf in Blueberry

The CPPU plays an important role in the callus subculture of blueberry leaves in five groups of the experiment (Table 10). As shown in Table 10, blueberries showed a different coefficient of increment and states on the medium with different concentrations of CPPU hormone. Under the combination of 3.0 mg/L CPPU, the increment coefficient of the leaf callus was the highest and reached 2.4.

**Figure 2:**
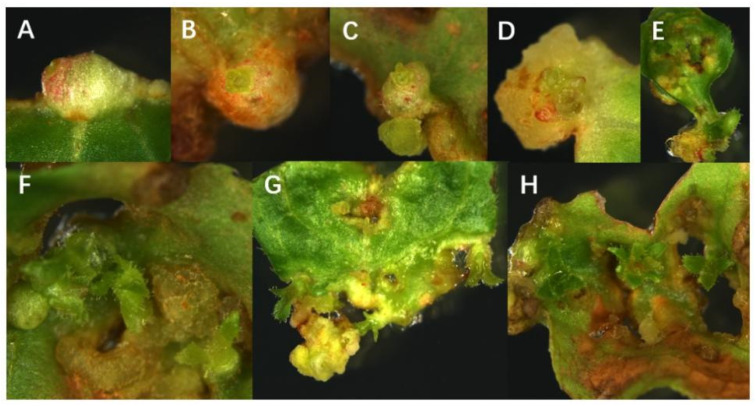
Phenotype in callus induction and bud differentiation from leaves in blueberry by the stereo microscope. (**A**) Emergence of bud primordium; (**B**–**D**) growth of bud primordium; (**E**) the appearance of buds; (**F**–**H**) clustered shoots. The bud in H can grow into a complete plantlet after culture. The picture was examined under magnification of 6.3 folds.

To further observe the bud induction process and state of the callus in blueberry leaves, a stereo microscope (Leica M205C) was used to observe the phenotype and status of the callus differentiated into buds under the callus induction medium culture. The results showed that the whole process of callus differentiation into buds could be clearly observed under 3.0 mg/L CPPU conditions (Figure 2).

#### 3.1.3. Bud from Callus Growth and Culture in Blueberry

**Figure 3:**
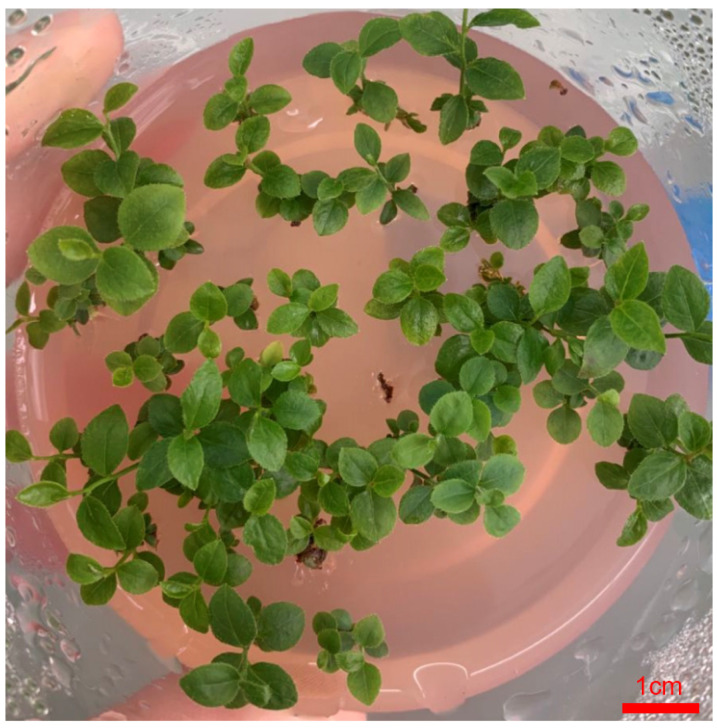
The buds grow to become tissue culture seedlings in blueberry. Bar, 1 cm.

Similarly, Table 11 showed that CPPU and 2-ip play an important role in differentiating between leaves and buds in blueberries. The result showed that leaves were differentiated into buds with large and bright green leaves, which had a strong and fast-growing state under the hormone combination of 1.0 mg/LCPPU and 0.4 mg/L 2-ip conditions (Figure 3). The condition was most suitable for the proliferation and propagation in blueberries (Figure 3).

#### 3.1.4. Root from Bud Growth and Culture in Blueberry

**Figure 4:**
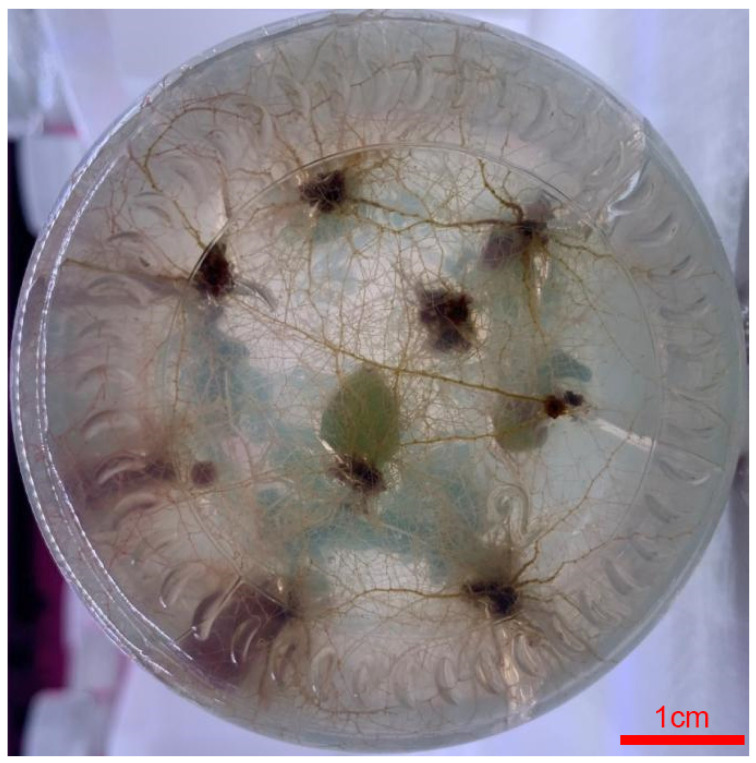
Phenotype of roots based on leaves under rooting medium in blueberries. Bar, 1 cm.

In the induction of the root test, a total of four treatments were performed (Table 12). As can be seen from Table 12, among the four treatments, treatment 3 had the best effect with significant differences and flourishing growth. Furthermore, the status of growth showed that the rooting time was short, the rooting rate reached more than 90%, and the adventitious roots were abundant and developed (Table 12). In summary, 2.0 mg/L NAA showed the best rooting effect on blueberries (Figure 4).

### 3.2. Regeneration System of Stem

#### 3.2.1. Pre-Culture of the Stem in Blueberry

**Figure 5:**
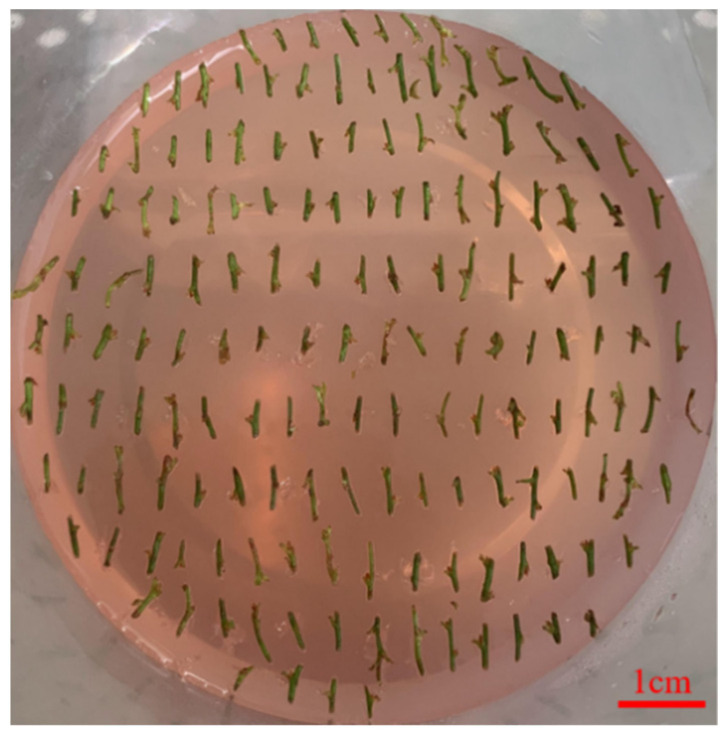
Germination state of the blueberry stem under dark conditions. Bar, 1 cm.

Rapid propagation by the stem in vitro is the key method to achieve large-scale and standardized cultivation of seedlings in blueberries. Therefore, the suitable tissue culture system is a key role in the propagation of seedlings in blueberries. The germination rate of the stem and the status of the bud after germination were calculated (Table 13). The statistical results showed that the optimal condition for blueberry stem growth in vitro was 0.2 mg/L IAA, 0.1 mg/L CPPU and 100 mg/L NaCl. The germination rate of the stem was 93%, and the germination state was the best (Figure 5, Table 13).

#### 3.2.2. Culture of the Proliferation of Stem in Blueberry

**Figure 6:**
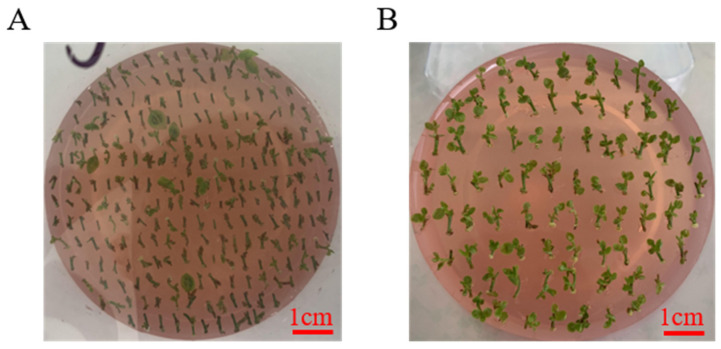
The phenotype of proliferation from the stem in blueberry under rapid culture medium. The phenotype of stem cultured for 5 days (**A**) and 13 days (**B**) in blueberry under rapid culture medium. Bar, 1 cm.

The pre-cultured stem in Section 3.2.1 was transferred to the medium of proliferation of the bud. In the study, the MS medium containing MES was used as the base medium, which contained ZT, putrescine, spermidine and spermine. The statistical results confirmed that the optimal condition for the proliferation of the blueberry stem in vitro was 0.4 mg/L ZT, 1 mg/L putrescine, 1 mg/L spermidine and mg/L spermine (Figure 6, Table 14). Further morphological analysis showed a fast proliferation rate in the adventitious buds with a vigorous growth status (Table 14, Figure 6). Adventitious buds could grow in most blueberry stems after 5 days, which gradually grew larger with time. After 13 days, most of the adventitious buds on the stem were formed to meet the need of rapid propagation (Figure 6).

#### 3.2.3. Growth Culture of Blueberry

**Figure 7:**
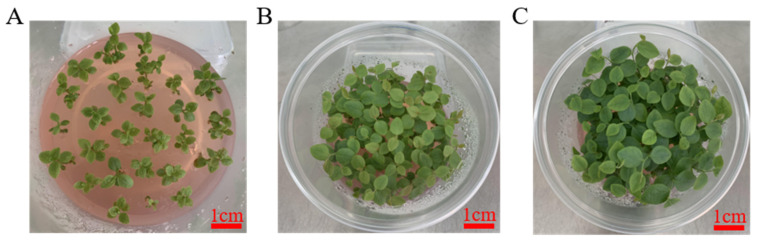
The phenotype of growth of plantlet from stem in blueberry under medium for culturing seedling. State of plantlet from stem cultured for 0 day (**A**), 10 days (**B**) and 10 days (**C**) in blueberries. Bar, 1 cm.

The blueberry stem in Section 3.2.3 was cultured for 13 days and transferred to five kinds of blueberry growth culture medium for 20 days. The result in Table 15 showed that blueberries have strong growth with light green leaves under MS medium containing MES, which contained 0.5 mg/L isopentene adenine (Figure 7). After 14 days of culture, the blueberry seedlings grew well and healthily, which met the need of rooting and propagating in blueberry seedlings (Figure 7).

#### 3.2.4. Rooting Culture in Blueberry

**Figure 8:**
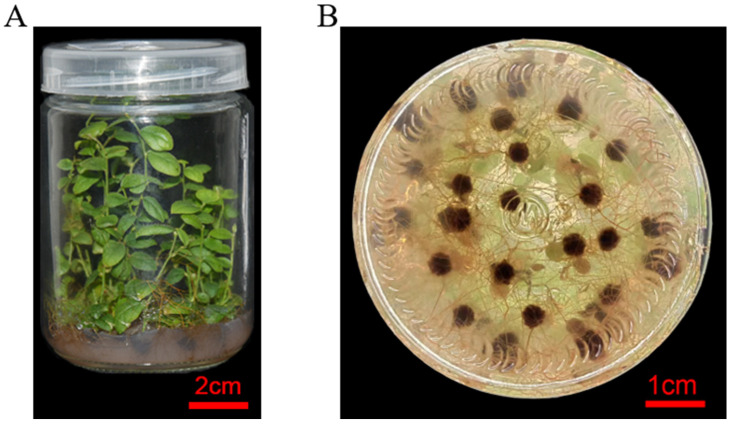
The phenotype of root in blueberry under medium for rooting culture of stem. The phenotype of plantlet (**A**) and root (**B**) for 35 days in blueberry. Bar, 1 cm.

The thriving blueberry seedlings in Section 3.2.3 were cut into 2–3 cm segments and then transferred to the rooting medium for rooting culture. The result showed that the concentration of high NAA inhibits the rooting, while the concentration of low NAA fails to achieve the rooting (Table 16). Moreover, the results in Table 16 show that the blueberry seedlings have more adventitious roots with strong growth, which have a shorter time for rooting (Figure 8). Therefore, the rooting culture could meet the needs of large-scale production in blueberries.

#### 3.2.5. Domestication and Large-Scale Production of Rooting Seedling

**Figure 9:**
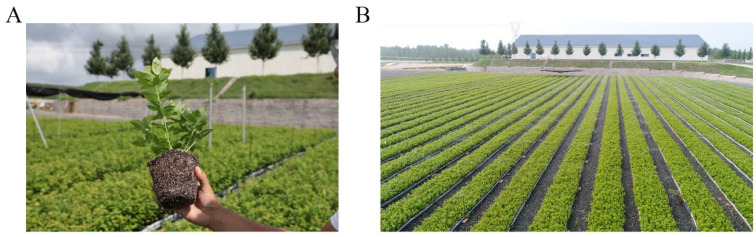
State of domestication (**A**) and large-scale production (**B**) in blueberries.

The rooted tissue culture seedlings were transplanted to the seedling bed after nursing of the plantlet. Subsequently, these seedlings could grow for about 5 weeks under moisture and heat preservation conditions. Subsequently, the blueberry seedlings were cultured by the integrated system of water and fertilizer to maintain growth under natural conditions. The result showed that the survival rate of blueberry seedlings was more than 90% (Figure 9). In general, the stable and efficient culture system in blueberries in the study can meet the need of large-scale and standardized production in the blueberry industry.

## 4. Discussion

Plant tissue culture has a short growth cycle and a high reproduction rate. The culture condition of plant tissue culture can be artificially controlled and automatically managed, which is conducive to the realization of factory management. Therefore, plant tissue culture is widely used in the large-scale production of blueberry seedlings. However, the stable and sustainable tissue culture system of blueberries is still lacking. Plant growth regulators play an important role in regulating the physiological structure, growth, development and morphogenesis in plant tissue culture. Therefore, it is the basis of plant tissue culture to study the appropriate hormone types and concentrations in plant regeneration [30,31]. Forchlorfenuron (CPPU) and 2-isopentenyladenine (2iP) play a key role in callus induction and regeneration in plants [32,33,34,35]. However, the effects of CPPU and 2ip on the generation and regeneration of the callus are rarely reported in blueberry leaves.

The study showed that 2.0 mg/L CPPU and 0.2 mg/L 2-ip can promote callus induction, and 3.0 mg/LCPPU can promote the growth of embryogenic callus in blueberry leaves (Figure 1 and Figure 2; Table 9 and Table 10). In addition, CPPU [36,37,38] and 2iP [39,40,41] are active and promote bud growth in blueberries. In the present experiment, 1.0 mg/LCPPU and 0.4 mg/L 2-ip promote bud production and growth in blueberry, and the growth state of buds is better (Figure 3; Table 11). Furthermore, growth is strong and fast in buds, and the leaves are verdant (Figure 3; Table 11). In addition, root architecture and growth play crucial roles in plant ecosystems [42,43], which cope with adverse living conditions [44,45,46]. For woody plants, it is widely known that auxin plays a key role in root formation at the bases of buds and stem cuttings [47,48,49]. The study showed that 2.0 mg/L NAA promotes root formation, and the rooting rate reached 90% (Figure 4; Table 12). For woody plants, cutting is critically important in clonal propagation [50]. Here, we determined that the regeneration system of blueberries was established based on the stems. Previously, Raldugina et al. (2021) [51] demonstrated that the NaCl interferes with the regeneration process in plants [52]. Meanwhile, IAA and CPPU play an essential role in stem propagation [53,54]. In the study, 0.2 mg/L IAA, 0.1 mg/L CPPU and 100 mg/L NaCl activated regeneration of stems in the pre-culture stage (Figure 5; Table 13) In addition, Polyamines (PAs) participate in many plant growth and developmental processes [55]. Furthermore, regeneration and propagation in plants were induced by zeatin [56]. The result showed that 0.4 mg/L zeatin, 1 mg/L putrescine, 1 mg/L spermine and 1 mg/L spermidine promoted propagation of blueberry stems (Figure 6; Table 14). In addition, cytokinin is an adenine derivative with an isoprenoid side chain, which plays a key role in plant growth and development [57,58]. Similarly, 0.5 mg/L isopentenyl adenine promotes the growth of the stem (Figure 7; Table 14). In the study, 2 mg/L IAA promoted root formation and growth (Figure 8; Table 16). Many reports can be found in Arabidopsis and poplar [59,60]. In general, there are many difficulties in technology transfer from laboratory to industrial and scale production in blueberries. The study established an integral and stable system in the large-scale production of blueberry seedlings. In conclusion, the study established a regeneration system based on leaf and stem, while providing a new idea and method in the establishment of a regeneration system, genetic engineering and industrial production in blueberries.

## 5. Conclusions

Plant tissue culture plays a critical role in vegetative propagation in blueberries. In the study, we established a high-efficiency tissue culture system based on the leaves and stems in blueberries, respectively. Based on leaves, the optimal medium of callus induction, secondary culture of leaf callus, bud growth and root growth were acquired in the study, respectively. In addition, we acquired preculture, fast cultivation, growth of plantlet and rooting medium based on the stem, which was the optimum growth and development in blueberries. In conclusion, our result established an efficient regeneration system based on leaves and stems in blueberries, which provided a solid foundation for the genetic improvement of blueberries and strong evidence that the precision breeding in blueberries and the standardization and industrialization of the blueberry industry.

## Figures and Tables

**Table 1:** Combination and concentration of hormones in leaf callus-inducing medium.

Treatment	Different Hormone Combinations
1	0.5 mg/L CPPU + 0.1 mg/L 2-ip
2	0.5 mg/L CPPU + 0.2 mg/L 2-ip
3	0.5 mg/L CPPU + 0.3 mg/L 2-ip
4	1 mg/L CPPU + 0.1 mg/L 2-ip
5	1 mg/L CPPU + 0.2 mg/L 2-ip
6	1 mg/L CPPU + 0.3 mg/L 2-ip
7	2 mg/L CPPU + 0.1 mg/L 2-ip
8	2 mg/L CPPU + 0.2 mg/L 2-ip
9	2 mg/L CPPU + 0.3 mg/L 2-ip
10	3 mg/L CPPU + 0.1 mg/L 2-ip
11	3 mg/L CPPU + 0.2 mg/L 2-ip
12	3 mg/L CPPU + 0.3 mg/L 2-ip
13	4 mg/L CPPU + 0.1 mg/L 2-ip
14	4 mg/L CPPU + 0.2 mg/L 2-ip
15	4 mg/L CPPU + 0.3 mg/L 2-ip
16	0.5 mg/L TDZ + 0.5 mg/L IBA
17	0.5 mg/L TDZ + 0.5 mg/L IAA
18	0.5 mg/L TDZ + 0.5 mg/L NAA

**Table 2:** The concentration of CPPU in subculture of callus medium.

Treatment	CPPU (mg/L)
1	0.5
2	1.0
3	2.0
4	3.0
5	4.0

**Table 3:** Combination and concentration of hormones in the bud growth of the leaf medium.

Treatment	CPPU (mg/L)	2-ip (mg/L)
1	0.5	0.4
2	0.5	0.6
3	1.0	0.4
4	1.0	0.6

**Table 4:** Concentration of NAA in rooting medium of bud from leaf.

Treatment	NAA (mg/L)
1	0.5
2	1.0
3	2.0
4	3.0

**Table 5:** Combination and concentration of hormones and substances in pre-culture medium of the stem.

Treatment	Different Hormone Combinations	Sucrose g/L	Agar g/L	NaCl Concentration mg/L
1	0.2 mg/L IAA + 0.1 mg/L CPPU	20	7	-
2	0.2 mg/L IAA + 0.5 mg/L CPPU	20	7	-
3	0.4 mg/L IAA + 0.1 mg/L CPPU	20	7	-
4	0.4 mg/L IAA + 0.5 mg/L CPPU	20	7	-
5	0.2 mg/L IAA + 0.1 mg/L CPPU	10	7	-
6	0.2 mg/L IAA + 0.1 mg/L CPPU	10	7	50 mg/L
7	0.2 mg/L IAA + 0.1 mg/L CPPU	10	7	100 mg/L
8	0.2 mg/L IAA + 0.1 mg/L CPPU	10	7	200 mg/L

**Table 6:** Combination and concentration of hormones and substances in the rapid medium of the stem.

Treatment	The Concentration of ZT	Concentration Putrescine, Spermidine and Spermine
1	0.2 mg/L	-
2	0.4 mg/L	-
3	0.4 mg/L	1 mg/L putrescine
4	0.4 mg/L	1 mg/L spermidine
5	0.4 mg/L	1 mg/L spermine
6	0.4 mg/L	1 mg/Lputrescine, 1 mg/Lspermidin, 1 mg/Lspermine

**Table 7:** The concentration of isopentene adenine in the culturing seedling medium of the stem.

Treatment	Concentration of Isopentene Adenine
1	0.1 mg/L
2	0.5 mg/L
3	1.0 mg/L
4	1.5 mg/L
5	2.0 mg/L

**Table 8:** The concentration of NAA in the rooting medium of the stem.

Treatment	NAA (mg/L)
1	0.5
2	1.0
3	2.0
4	3.0

**Table 9:** Phenotype and statistics of leaf-induced callus under callus-inducing medium.

Treatment	Time of Callus Appearance	The Status of the Callus	The Color of the Callus	Callus Induction Rate	Callus Differentiation Rate
1	About 7 d	Compaction, small spherical form	White	44%	19%
2	7–9 d	Compaction	White or light green	67%	17%
3	6–10 d	Compaction, the surface has obvious granulum	light green	55%	10%
4	About 7 d	Compaction, spherical	White	66%	38%
5	About 7 d	Compaction, spherical	White	72%	35%
6	About 7 d	Compaction, spherical, granulum	light green	72%	29%
7	5–9 d	Compaction, spherical	light yellow	91%	66%
8	5–9 d	Compaction, Spherical surface with fewer small granulum	White or light yellow	97%	71%
9	5–9 d	Compaction, spherical,	White or light green	97%	54%
10	5–9 d	Compaction, spherical	White	95%	49%
11	5–9 d	Compaction, Spherical surface with fewer small granulum	White or light yellow	93%	52%
12	5–9 d	Compaction, spherical	White or light yellow	98%	44%
13	5–9 d	Compaction, spherical	White or light yellow	93%	36%
14	5–9 d	Compaction, Spherical surface with fewer small granulum	White or light yellow	95%	27%
15	5–9 d	Compaction, spherical	White or light yellow	96%	21%
16	8–10 d	Compaction, slightly transparent	White or light yellow	74%	15%
17	8–10 d	Compaction, slightly transparent, particle	White or light yellow	82%	23%
18	About 10 d	small particle	White	5%	0%

**Table 10:** Statistics of subculture in callus based on leaf.

Treatment	Multiplication Coefficient
1	0.9
2	1.4
3	1.8
4	2.4
5	1.6

**Table 11:** Phenotype of bud growth based on leaf.

Treatment	Status of Bud Growth
1	Poor, slow, small leaves
2	General, slower, moderate
3	Good, strong and fast, larger leaf
4	General, slower, moderate

**Table 12:** Phenotype and statistics of in rooting rate based on bud of leaf.

Treatment	Rate of Rooting (%)	Status of Growth
1	69%	Slow rooting
2	76%	more adventitious roots
3	90%	The rooting time was shorter and the adventitious roots were more
4	85%	The rooting time was shorter and the adventitious roots were more

**Table 13:** Statistics and phenotype of stem under pre-culture medium.

Treatment	Germination Rate of Stem	The Status of Bud after Germination
1	68%	Good, fast growth
2	64%	General
3	57%	General
4	49%	Poor, slow growth
5	67%	Good, fast growth
6	85%	Good, fast growth, mild stress
7	93%	Good, fast growth; mild stress
8	43%	Poor, slow growth; severe stress

**Table 14:** The phenotype for the proliferation of the stem under a rapid culture medium.

Treatment	Status of Growth
1	General, slow growth
2	Good, slow growth
3	Good, slow growth
4	General, slow growth
5	Good, slow growt
6	Good, fast growth

**Table 15:** The phenotype for status of seedling based on stem.

Treatment	Growth Status of Seedling
1	Poor growth, slower growth, plant is weak, small leaves
2	Better growth, plant is stronger, large and light green leaves
3	Good growth, the plant is strong
4	Good growth, the plant is stronger
5	Poor growth, slower growth, plant is weak, small leaves

**Table 16:** Statistics and phenotype for roots based on stems under rooting medium.

Treatment	Rooting Rate (%)	Growth State
1	68%	Slow rooting
2	71%	More adventitious root
3	93%	Shorter rooting time, more adventitious root
4	83%	Shorter rooting time, more adventitious root

## Data Availability

Not applicable.
